# P800SO3-PEG: a renal clearable bone-targeted fluorophore for theranostic imaging

**DOI:** 10.1186/s40824-022-00294-2

**Published:** 2022-10-01

**Authors:** Haoran Wang, Homan Kang, Jason Dinh, Shinya Yokomizo, Wesley R. Stiles, Molly Tully, Kevin Cardenas, Surbhi Srinivas, Jason Ingerick, Sung Ahn, Kai Bao, Hak Soo Choi

**Affiliations:** 1 Wuya College of Innovation, Shenyang Pharmaceutical University, Shenyang, China; 2 Gordon Center for Medical Imaging, Department of Radiology, Massachusetts General Hospital and Harvard Medical School, Boston, MA USA

**Keywords:** NIR imaging, Targeted fluorophore, Structure-inherent targeting, Bone targeting, Renal clearance

## Abstract

**Background:**

Due to the deep tissue penetration and reduced scattering, NIR-II fluorescence imaging is advantageous over conventional visible and NIR-I fluorescence imaging for the detection of bone growth, metabolism, metastasis, and other bone-related diseases.

**Methods:**

Bone-targeted heptamethine cyanine fluorophores were synthesized by substituting the *meso*-carbon with a sulfur atom, resulting in a bathochromic shift and increased fluorescence intensity. The physicochemical, optical, and thermal stability of newly synthesized bone-targeted NIR fluorophores was performed in aqueous solvents. Calcium binding, bone-specific targeting, biodistribution, pharmacokinetics, and 2D and 3D NIR imaging were performed in animal models.

**Results:**

The newly synthesized S-substituted heptamethine fluorophores demonstrated a high affinity for hydroxyapatite and calcium phosphate, which improved bone-specific targeting with signal-background ratios > 3.5. Particularly, P800SO3-PEG showed minimum nonspecific uptake, and most unbound molecules were excreted into the urinary bladder. Histological analyses demonstrated that P800SO3-PEG remained stable in the bone for over two weeks and was incorporated into bone matrices. Interestingly, the flexible thiol ethylene glycol linker on P800SO3-PEG induced a promising photothermal effect upon NIR laser irradiation, demonstrating potential theranostic imaging.

**Conclusions:**

P800SO3-PEG shows a high affinity for bone tissues, deeper tissue imaging capabilities, minimum nonspecific uptake in the major organs, and photothermal effect upon laser irradiation, making it optimal for bone-targeted theranostic imaging.

**Supplementary Information:**

The online version contains supplementary material available at 10.1186/s40824-022-00294-2.

## Introduction

Noninvasive imaging technology allows for the visualization of bone growth, abnormalities, and metabolism, while playing an integral role in image-guided interventions and surgical procedures of the bones [1–3]. Traditional bone imaging modalities such as X-ray radiogrammetry, computed tomography, quantitative ultrasound, position emission tomography, and magnetic resonance imaging, are routinely used for the inspection of bone fractures, injuries, and joint abnormalities [4]. Furthermore, the advancement in optical imaging systems and contrast agents enables us to view the detailed changes in bones with high spatiotemporal resolution wide-field imaging [5–12].

Near-infrared (NIR) fluorescence imaging is an emerging technology well suited to studying the functional and structural aspects of bone diseases due to the reduced tissue absorption and scattering of NIR light and minimum autofluorescence [13–15]. Compared to the traditional NIR imaging (NIR-Ia, 650–900 nm), the NIR-II window (1,000–1,700 nm) is more attractive due to its ability for deeper light penetration and lower tissue scattering, resulting in enhanced signal-to-background ratios (SBR) [16–18]. It is worth noting that the classical NIR-I fluorophore, indocyanine green (ICG), has been explored for NIR-II tail imaging of various cancerous tissues in the clinic [19–21]. Due to the finer structure of bone and the inherent limitations of conventional imaging modalities, NIR-II imaging has the potential to obtain high-resolution bone diagnostics for use in clinical applications.

**Fig. 1:**
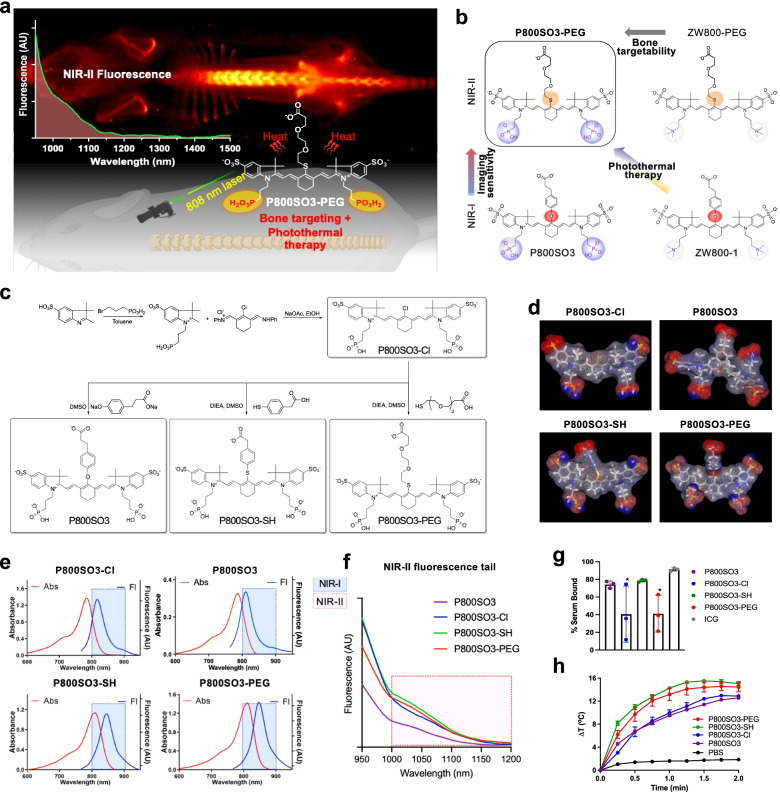
Chemical design and optophysical properties of bone-targeted theranostic imaging. **a** P800SO3-PEG is a bone-targeted heptamethine cyanine fluorophore for NIR-II tail imaging. **b** Design of P800SO3-PEG using the structure-inherent targeting strategy. **c** Synthetic routes for bone-targeted agents. **d** 3D energy minimized chemical structures of the designed fluorophores. **e**, **f** Absorbance (Abs) and fluorescence (Fl) spectra of bone-targeted agents in the NIR-1 (**e**) and NIR-II fluorescence tail imaging (**f**) recorded for P800SO3-Cl, P800SO3, P800SO3-SH, and P800SO3-PEG (λ_ex_ = 760 nm) in 10% fetal bovine serum (FBS) solution. **g** Plasma protein binding assay of bone-targeted NIR fluorophores compared with ICG incubated in 5% bovine serum albumin (BSA)-containing saline for 4 h. **h** Photothermal effect of bone-targeted NIR fluorophores in PBS (30 µM) under continuous 808 nm exposure for 2 min at a power density of 1.0 W/cm^2^

However, unfortunately, there is no bone-specific NIR-II imaging probe that enables real-time noninvasive detection of bone growth and tissue microcalcification. Here we designed targeted NIR fluorophores for noninvasive imaging of bone tissue based on the structure-inherent targeting (SIT) strategy, in which targeting moieties or pharmacophores are incorporated into the chemical structure of a fluorophore [22]. We modified previously developed NIR fluorophores to generate a renal clearable bone-targeted contrast agent with improved optical properties and biodistribution, in addition to enabling a photothermal effect (Fig. 1a). By introducing a sulfur atom to the *meso*-carbon, the peak emission wavelength of the final fluorophore is shifted into the NIR-II ranges with increased sensitivity to light irradiation.

## Methods

### Materials

All chemicals and solvents were of American Chemical Society or HPLC purity and were used as received. HPLC grade methanol (MeOH), ethanol (EtOH), acetonitrile (ACN) and distilled water (DW) were purchased from Fisher Scientific (Pittsburgh, PA). All other chemicals, including dimethyl sulfoxide (DMSO), dimethylformamide (DMF), ethyl acetate (EA), and *N,N*-diisopropylethylamine (DIEA), were purchased from Fisher Scientific (Pittsburgh, PA, USA) and Sigma-Aldrich (St. Louis, MO). The purity of all of the compounds was measured using liquid chromatography-mass spectrometry (LC–MS), which consisted of an Alliance e2695 separation module (Waters), a 2998PDA detector (Waters, 212–800 nm), and an Acquity QDA detector (Waters, m/z range: 50–1,239). An XBridge C18 (4.6 × 150 mm, 5 µm) reversed-phase HPLC column (Waters) was used for LC–MS. The final compound was purified using Preparative HPLC consisting of a Waters 2489 UV/Visible detector and a Waters 1525 Binary HPLC pump, employing an XBridge Prep C18 (19 × 150 mm, 5 µm) reversed-phase HPLC column (Waters).

### Optical and physicochemical property analyses

All optical measurements were performed at 37 °C in 10% fetal bovine serum (FBS). The partition coefficient (logD at pH 7.4) of each fluorescent conjugate was calculated using JChem calculator plugins (ChemAxon, Budapest, Hungary). Fluorescence quantum yield (QY) was measured for each fluorescent conjugate using ICG in FBS (QY 10.1%) as a calibration standard under conditions of matched absorbance at 765 nm. Absorbance and fluorescence spectra for all fluorescent conjugates were collected using USB2000 + VIS–NIR-ES and USB2000FL fluorescence (350–1,000 nm) spectrometers (Ocean Insight, Dunedin, FL) for NIR-I spectra and Flame NIR-II spectrofluorometer for NIR-II spectra (950–1,650 nm). NIR-I excitation was provided by a 760 nm NIR laser diode light source (Electro Optical Components, Santa Rosa, CA), while an 808 nm NIR laser diode light was used for NIR-II excitation (Nawoo Vision, S. Korea).

### Physicochemical and photostability

To elucidate the relationship between the stability and concentration, P800SO3-Cl and P800SO3-PEG were dissolved in DI water. The maximum solubility of P800SO3-Cl was found to be about 60 mg/mL, while P800SO3-PEG shows an improved solubility in water (> 100 mg/mL) due to the introduction of a flexible PEG linker. Then, working solutions were prepared with various concentrations from 10 to 250 µM in 5% bovine serum albumin (BSA) in saline. For photostability, 200 µL of working solutions were irradiated using a continuous 808 nm laser diode at 35 mW/cm^2^ and imaged every 30 min for 120 min. Stability was determined by measuring the regions of interest (ROI) and comparing the fluorescence intensity against the starting fluorescence signal. To measure the photothermal effect, 30 µM of ICG, P800SO3, and P800SO3-PEG in 1X PBS solution were prepared from 10 mM dye stock solutions in DMSO. PBS was used as a control. 500 µL of working solutions and blank PBS in 1 mL cuvettes were irradiated using an 808 nm laser diode at 1 W/cm^2^ for 2 min, and imaged using a thermal camera (Xintest, HT-19). Thermal readings were recorded every 15 s for each fluorophore.

### Electron density calculations

Electron density calculations were performed using Gaussian 16 (Gaussian.com). The geometry optimizations of each molecule were performed using the density functional theory (DFT) with Becke’s three-parameter hybrid exchange function with Lee–Yang–Parr gradient-corrected correlation functional (B3-LYP functional) and 6-31G(d) basis set. Solvent effects in water were calculated using the polarizable continuum model (PCM) method. No constraints to bonds/angles/dihedral angles were applied in the calculations, and all atoms were free to optimize. The Mulliken charge [23] at each atom was recorded, after which the output file (.log) was imported into Avogadro 1.2.0. The electron density at isovalue = 0.2, and the electrostatic potential at the surface was calculated. Analysis using the Mulliken charge and the electrostatic potential (ESP) on the electron density surface shows that the overall electron density on the heptamethine chain of P800SO3-PEG is higher than P800SO3.

### Serum binding assay

Working solutions with a concentration of 10 µM in 100% mouse plasma were prepared from 10 mM dye stock solutions in DMSO and added into the assigned sample chambers of a Rapid Equilibrium Dialysis (RED) Device (Thermo Fisher, Waltham, MA) along with PBS as the dialysis buffer. The device was covered and incubated at 37 °C on an orbital shaker at 20 rpm for 4 h. After incubation, the absorbance and fluorescent profiles of each fluorophore were measured using NanoenTek JuLi Stage to calculate the concentrations of dyes in both chambers separately. The percentage of free and bound dye was calculated for each fluorophore, and data (*n* = 3) are presented as means ± standard deviation (S.D.).

### Calcium salts binding assay

Calcium carbonate, calcium oxalate, hydroxyapatite, calcium phosphate, and calcium pyrophosphate salts (25 mg/mL) were incubated separately with 5 µM working solutions in 1 mL saline. The calcium salts were vortexed continuously with the fluorophores at room temperature for 30 min. The mixtures were then washed 3 times with saline, followed by centrifugation at 3,000 rpm for 10 min to remove the unreacted fluoroprobes. To compare the binding affinities, the collected precipitate after centrifugation was dispersed in 200 μL of saline, and the fluorescence imaging system determined the fluorescence intensities of the dispersed samples. All NIR fluorescence images were collected at identical exposure times and are displayed with equal normalization.

### Microscope imaging

5 mg/mL of various calcium salts were vortexed in a 5 μM aqueous solution of P800SO3-PEG (1 mL) for 30 min at room temperature. The mixture was centrifuged and washed twice with 1X PBS. A portion of the residual powder was soaked in 1X PBS (0.5 mL) and then added into a 24-well plate. A customized 4-channel Nikon TE2000 epifluorescence microscope was used to determine the fluorescence intensities of the dispersed samples. The fluorescence intensity of each cell was measured using ImageJ. All NIR fluorescence images were collected at identical exposure times and are displayed with equal normalization.

### NIR fluorescence imaging systems

NIR-I images were obtained using the K-FLARE imaging system as described previously in detail [24–27]. Briefly, a 760 nm laser source (11 mW/cm^2^) was used for excitation, and a 785 nm long-pass (LP) filter was used to collect the NIR-I signal. White light (400–650 nm; 40,000 lx) was employed along with NIR imaging to acquire color and NIR fluorescence images simultaneously with customized software. For NIR-II imaging, a 640 × 512-pixel InGaAs camera (Ninox640, Raptor Photonics) and a macro zoom lens (0–10 × ; Navitar Zoom 7000 with SWIR coating) were assembled along with an 808 nm fiber-coupled laser excitation light (35 mW/cm) and a 1,070 nm LP filter (Midwest Optical Systems) to collect the NIR-II fluorescence signal. The imaging head was positioned at a distance of 15–25 cm from the surgical field, and all NIR fluorescence images had identical exposure times and normalizations [28].

### Animal models

Animals were housed in an AAALAC-certified facility, and all procedures were performed according to the approved institutional protocol by MGH (protocol #2016N000136). CD-1 mice (6–8 weeks, male) were purchased from Charles River Laboratories (Wilmington, MA). Athymic NCr *nu/nu* mice (6 weeks) were purchased from Taconic Biosciences (Rensselaer, NY). Mice were given free access to food and water and fed with chlorophyll-free mouse chow (VWR International, Radnor, PA) 5 d prior to the imaging study to minimize autofluorescence. To investigate the biodistribution and clearance of each NIR fluorophore, 0.25–1.0 mM of working solutions were prepared in 5% bovine serum albumin (BSA)-containing saline from 10 mM stock solutions in DMSO. Then, 100 µL (25–100 nmol) of each NIR fluorophore was intravenously injected via the retro-orbital sinus under isoflurane anesthesia 4 h prior to imaging. Animals were sacrificed after intraoperative NIR fluorescence imaging, and the major organs, including heart, lung, liver, pancreas, spleen, kidney, duodenum, intestine, and muscle, were excised and imaged for tissue-specific biodistribution. The fluorescence intensity of a region of interest (ROI) over each organ/muscle was quantified using ImageJ version 1.52p. The signal-to-background ratio (SBR) was calculated as SBR = target signal/muscle signal.

### 3D fluorescence tomography imaging

Mice were imaged in the InSyTe FLECT/CT system (TriFoil Imaging, Chatsworth, CA). CT was performed using the following parameters: tube voltage 31 kV, tube current 500 µA, and exposure time 150 ms. The entire object was scanned according to the principle of continuous helical radiation of 720 projections with 360-degree coverage. Image resolution reached 25 × 25 × 25 microns. The total time of the procedure was 15 min. FLECT was performed using the 780 nm laser, and the fluorescence signal was filtered with 853 nm filter emission. Exposure time was 17.5 ms. The entire object was scanned according to the principle of continuous helical radiation of 116 projections with 360-degree coverage. Image resolution reached 1 mm × 1 mm × 1 mm. The total time of the procedure was 33 min 12 s. The images were reconstructed using the TriFoil Imaging software. CT and FLECT reconstructions were combined using VivoQuant v2.5 (Invicro, Boston, MA).

### Quantitative analysis

At each time point, the fluorescence and background intensities of a region of interest (ROI) over each tissue were quantified using customized software. The signal-to-background ratio (SBR) was calculated using ImageJ version 1.52p as SBR = fluorescence/background, where background is the signal intensity of neighboring tissues, such as muscle or skin, obtained over the imaging period. All NIR fluorescence images for a particular fluorophore were normalized identically for all conditions of an experiment. At least three animals were analyzed at each time point. Statistical analysis was carried out using the unpaired Students t-test or one-way analysis of variance (ANOVA). Results are presented as mean standard deviation, and curve fitting was performed using the Prism version 9.2.0 software (GraphPad, San Diego, CA).

### Histology and NIR fluorescence microscopy

To determine the tissue distribution of the NIR fluorophore, bone tissues were removed from CD-1 mice 1 day and 14 days post-injection with 50 nmol of P800SO3-PEG in 100 µL of 5% wt/v BSA/saline. The dissected tissues were trimmed and embedded in Tissue-Tek optimum cutting temperature (OCT) compound (Sakura Finetek, Torrance, CA) without a pre-fixation step, and the tissue block was frozen at -80 °C. Ten-µm thick frozen sections were cut by a cryostat (Leica, Germany). The slides were subject to fluorescence analysis first, then stained for hematoxylin and eosin (H&E). Fluorescence and brightfield images were acquired on the 4-channel Nikon TE2000 epifluorescence microscope. Image acquisition and analysis were performed using IPLab software (Scanalytics, Fairfax, VA). A custom filter set (Chroma Technology, Brattleboro, VT) composed of a 710 ± 25 nm excitation filter, a 785 dichroic mirror, and an 810 ± 20 nm emission filter were used for imaging. Exposure times were adjusted to obtain a similar maximum fluorescence value for each fluorescence image. Brightfield images of H&E-stained slides from a matching field of view were also obtained.

## Results

### Chemical design of bone-targeted NIR fluorophores

As shown in Fig. 1b, P800SO3-PEG was designed around P800SO3 [29] by substituting the phloretic acid linker with a serum-stable yet flexible thiol PEG linker, resulting in a bathochromic shift of the maximum absorption and emission spectra, thus enabling NIR-II tailing imaging. The phosphonate and sulfonate sidechains are retained at the same position, which together induces bone targetability compared to non-targeted ZW800-PEG [14, 29]. To compare the optical and functional properties, P800SO3-Cl was used to modify the *meso*-carbon of the heptamethine core with 4-mercaptophenylacetic acid (SH) and produced P800SO3-SH. P800SO3 was used as a control (Fig. 1c). Prior to the measurement of optical properties, each NIR fluorophore was purified by prep-HPLC to yield > 95% purity, measured by the HPLC–PDA at 254 nm absorbance (Fig. S1) and ^1^H and ^13^C NMR spectroscopy (Fig. S2), respectively. The 3D energy-minimized structures were calculated by MarvinSketch (ChemAxon, Budapest, Hungary) to compare their charge distribution and conformational differences (Fig. 1d). All the designed fluorophores display relatively rigid backbones without twisting the sidechains due to the cyclohexene in the middle of heptamethine core [30]. The short and rigid 4-mercaptophenylacetic acid on P800SO3-SH is positioned to face one of the sulfonates, increasing *meso*-steric hindrance between the aromatic ring and delocalized framework. Interestingly, the flexible nonaromatic thiol PEG linker of P800SO3-PEG faces straight upward, isolating the carboxylate from the untwisted backbone.

### Physicochemical properties of bone-targeted NIR fluorophores

The physicochemical properties of the NIR fluorophores were calculated by JChem (ChemAxon) and summarized in Table 1. Since the final NIR fluorophores are highly charged, the distribution coefficient log*D* is an essential predictor of lipophilicity and serum protein binding of the final compound in the blood. P800SO3-Cl is hydrophilic (Log*D* at pH 7.4 = -4.80) but can be made even more so by adding SH or PEG linker to the backbone, which decreases the log*D* to -7.04 and -8.65, respectively. Topological polar surface area (TPSA) is an indicator of tissue absorption and penetration, and by adding hydrophilic linkers to the *meso*-chlorine of P800SO3-Cl, the TPSA increased 40–60 Å^2^.

**Table 1:** Physicochemical properties of bone-targeted NIR fluorophores

**Fluorophore**	**P800SO3-Cl**	**P800SO3** [29]	**P800SO3-SH**	**P800SO3-PEG**
MW (Da)	857.15	986.23	988.19	1,014.23
LogD, pH 7.4	-4.80	-7.03	-7.04	-8.65
TPSA (Å^2^)	235.71	285.07	275.84	294.30
HBA/HBD	13/4	16/4	15/4	17/4
Charges (+/-)	1/4	1/5	1/5	1/5
Rotatable bonds	13	18	17	23

### Optical properties of bone-targeted NIR fluorophores

Table 2 summarizes the optical properties of bone-targeted NIR fluorophores measured in 10% fetal bovine serum (FBS). P800SO3-Cl emits maximum fluorescence at 817 nm with high extinction coefficient (ε = 275,200 M^−1^ cm^−1^) and quantum yield (QY; Φ = 9.28%). Oxygen substitution at the *meso*-carbon of the same heptamethine backbone resulted in a hypsochromic shift of fluorescence emission (λ_em_ of P800SO3 = 802 nm), while the sulfur atom replacement generated 42–45 nm bathochromic shifts (λ_em_ of P800SO3-SH = 845 and P800SO3-PEG = 847 nm), enabling NIR-II tail imaging (Fig. 1e,f).

**Table 2:** Optical properties of bone-targeted NIR fluorophores in 10% FBS

**Fluorophore**	**P800SO3-Cl**	**P800SO3** [29]	**P800SO3-SH**	**P800SO3-PEG**
λ_abs_ (nm)	783	785	810	810
λ_em_ (nm)	817	802	845	847
Stokes shift (nm)	34	17	35	37
ε (M^−1^ cm^−1^)	275,200	210,000	227,600	285,400
QY (Φ, %)	9.28	15.10	7.08	7.34
Brightness (M^−1^ cm^−1^)	25,539	31,710	16,114	20,948

### Plasma protein binding, photostability, and photothermal effect

Next, to find the serum stability of bone-targeted NIR fluorophores, we measured their plasma protein binding (PPB) using a Rapid Equilibrium Dialysis (RED) Device (Fig. 1g). NIR fluorophores were prepared at a concentration of 10 µM in 5% bovine serum albumin (BSA)-containing saline and incubated for 8 h at 37 °C with gentle shaking. ICG was used as a control. P800SO3-Cl and P800SO3-PEG showed around 40% PPB, while P800SO3 and P800SO3-SH bound significantly to plasma proteins (> 70%). These PPB data are useful to predict the biodistribution and clearance of injected doses in the body [31, 32]. We also measured the photostability of these NIR fluorophores at different concentrations (10–100 µM) by irradiating them using an 808 nm laser at a power density of 35 mW/cm^2^ for 2 h under the home-built NIR-II imaging system (Fig. S3a) [28]. P800SO3 and P800SO3-Cl showed relatively good photostability at concentrations > 50 µM. Then, we fixed the concentration of each fluorophore at 30 µM in phosphate-buffered saline (PBS) and irradiated the samples under an 808 nm laser for 2 min to prove our hypothesis that these NIR fluorophores display photothermal effects (Fig. 1h). Overall, elevated temperature changes (10–15 °C) were observed in all the tested NIR fluorophores upon laser irradiation. Specifically, P800SO3-PEG and P800SO3-SH displayed the fastest photothermal effects, which is coincident with their photosensitivity results in Fig. S3a. Compared to P800SO3, P800SO3-PEG showed a 2–4 °C higher temperature increase, probably due to the difference in electron density and steric hindrance in each polymethine chain.

### Physicochemical stability measurements

Thermal and pH stabilities of P800SO3-PEG were measured by preparing 1 mM and 5 µM working solutions in DI water, respectively. Fig. S3b, P800SO3-PEG shows reasonable thermal stability in a wide range of temperatures from -80 to 25 °C for 8 h post-incubation. It also displays pH stability from pH 1 to pH 9 (Fig. S3c). The heptamethine backbone is known to be unstable under strongly basic conditions [33–35]. Interestingly, the overall electron density on the heptamethine chain of P800SO3-PEG is higher than P800SO3 (Fig. S3d), because the π-donation from the sulfur atom (S) is greater than that of the oxygen (O) [36]. Upon light irradiation, P800SO3-PEG undergoes the oxidative addition of a reactive singlet oxygen species to the heptamethine chain, followed by decomposition to generate non-fluorescent keto fragments [37, 38].

### Calcium salt binding assay

**Fig. 2:**
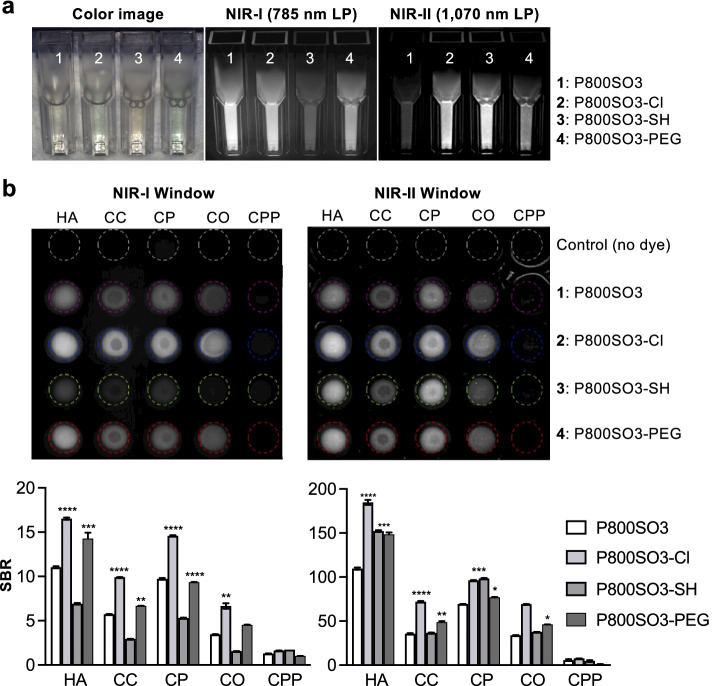
Calcium salt binding assay of the designed bone-targeted agents under NIR-I and NIR-II filters. 760 nm excitation and 785 nm LP filters were used for NIR-I imaging, and 808 nm excitation and 1,070 nm LP filters for NIR-II imaging. **a** 5 µM of each fluorophore in 10% FBS was imaged. **b** Calcium-salt binding assay of the bone-targeted agents. SBRs were calculated by dividing the fluorescence intensity of each fluorophore by the signal intensity of each blank (which include only calcium salt and PBS). Data is expressed as mean ± S.D. (*n* = 3): *p < 0.05, **p < 0.01, ***p < 0.001, ****p < 0.0001. HA, hydroxyapatite; CC, calcium carbonate; CP, calcium phosphate; CO, calcium oxalate; CPP, calcium pyrophosphate

We further measured the fluorescence emission tail of the NIR fluorophores in the NIR-I (Ex 760 nm, Em 785 nm longpass (LP)) and NIR-II (Ex 808 nm, Em 1,070 nm LP) windows (Fig. 2a). These fluorophores were then tested in vitro for their ability to bind biologically relevant calcium salts, including hydroxyapatite (HA), calcium carbonate (CC), calcium phosphate (CP), calcium oxalate (CO), and calcium pyrophosphate (CPP) (Fig. 2b) [39]. Among the five major calcium salts found in human body, HA is the major component and is also found in atherosclerotic plaques and bone neoplasms [40]. Generally, none of the fluorophores showed strong binding with CPP in the assay. Compared with the other three fluorophores, P800SO3-Cl showed stronger binding to HA in the NIR-I and NIR-II windows, but its binding difference between HA, CC, CP, and CO was minimal. Overall, the signal intensity of bone-targeted agents in the NIR-II window is tenfold higher than those in the NIR-I window. To find more detailed binding patterns and morphology with calcium salts, P800SO3-PEG was selected and observed under NIR microscopy. As shown in Fig. S4, strong signals were observed in HA, CP, and CO, but almost no signal was observed in CPP, indicating minimal binding, which is consistent with the in vitro data in Fig. 2b.

### Bone-specific targeting

**Fig. 3:**
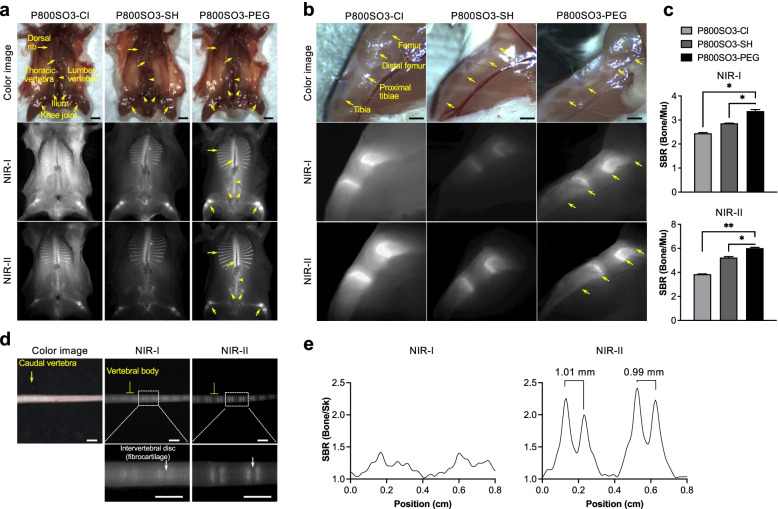
Biodistribution and bone-targeting of P800SO3-Cl, P800SO3-SH, and P800SO3-PEG in mice. 50 nmol (2.5 mg kg^−1^) of each NIR fluorophore was injected intravenously to 20 g CD-1 mice 4 h prior to imaging. Whole body bone imaging was conducted (**a**) as well as knee joint imaging (**b**) under color (top), NIR-I (middle), and NIR-II (bottom) channels. Scale bars = 5 mm (**a**) and 2 mm (**b**). **c** The SBR of each knee bone over the neighboring muscle tissue was calculated (*n* = 3, mean ± S.D): **p < 0.01, ***p < 0.001, ****p < 0.0001. **d** Caudal vertebrae were imaged under the NIR-I and NIR-II window 4 h post-injection of P800SO3-PEG. Scale bars = 3 mm. **e** FWHM analysis of the tail highlighted by the white dashed box in (**d**). Based on the cross-sectional intensity profiles in NIR-II, the length of the coccyx condyle spacings was 0.99–1.01 mm

To evaluate bone-specific binding along with the biodistribution and clearance, 50 nmol of each NIR fluorophore was administered intravenously to CD-1 mice and imaged under the NIR-I and NIR-II windows up to 4 h post-injection (Fig. 3). Prior to imaging, all internal organs were removed, and the supine posture was fixed. All the injected NIR fluorophores successfully highlighted the thoracic, lumbar, and sacral vertebrae, as well as the dorsal ribs, ilium, and knee joints of the mouse, with high fluorescence signals (Fig. 3a,b). Due to its relatively high LogD value and poor binding selectivity to HA, P800SO3-Cl exhibited elevated nonspecific uptake in the muscle and skin, resulting in the lowest SBR among the three (≈ 2.3 in NIR-I and ≈ 3.9 in NIR-II). On the other hand, P800SO3-SH shows overall low signals in the bone and background tissues due to the poor photostability at low concentrations (< 50 µM). P800SO3-PEG presented minimum background signals, and the entire bone structure was highlighted clearly. Consequently, the SBR of P800SO3-PEG in the bone under NIR-II (SBR ≈ 6.0, ***p < 0.0001 compared to P800SO3-Cl) was significantly higher than that of NIR-I (SBR ≈ 3.5, ***p < 0.001 compared to P800SO3-Cl) due to lower tissue scattering (Fig. 3c). Imaging of other bone types, including the spinal column vertebrae and ribs, were also compared in Fig. S5a,b, where P800SO3-PEG proved to have significant bone-specific uptake with an SBR ≈ 3.0 in the ribs and SBR ≈ 10 in the spine, respectively (***p < 0.001 compared to P800SO3-Cl).

Next, we compared P800SO3-PEG with P800SO3 using NIR-I and NIR-II cameras. As shown in Fig. S5c-f, P800SO3-PEG shows relatively high brightness comparable to P800SO3 under the NIR-I FLARE imaging system. However, the brightness of P800SO3-PEG was significantly enhanced under the NIR-II window in both bones of the thoracic vertebrae (**p <0.01; Fig. S5c) and spine (***p <0.001; Fig. S5d). The resected organs were also compared under the two imaging systems, where NIR-II imaging of P800SO3-PEG showed significantly higher SBR than NIR-I imaging (*p <0.05 or **p <0.01). Of note, since renal clearance is the main excretion route, the signal in the kidneys is 3-8 fold higher than that in the other organs. Overall, the SBR of bones in the NIR-II window was higher than in the NIR-I window due to reduced background signals and minimal scattering [41].

To compare the resolution of images under the two NIR windows, mouse tail bone imaging was performed. 50 nmol of P800SO3-PEG was injected 24 h prior to imaging, and tails with intact skin were located sequentially under the two imaging systems (Fig. 3d). Bone images under the NIR-II window are generally clearer and sharper with an average SBR of 2.4 which is about two-fold higher than that in the NIR-I window (SBR ≈ 1.3). The average full width at half maximum (FWHM) of the average intervertebral disc space was 1.00 ± 0.01 mm in the NIR-II window, however the Gaussian fit failed for the NIR-I image due to the increased blur (Fig. 3e).

### Biodistribution and pharmacokinetics

**Fig. 4:**
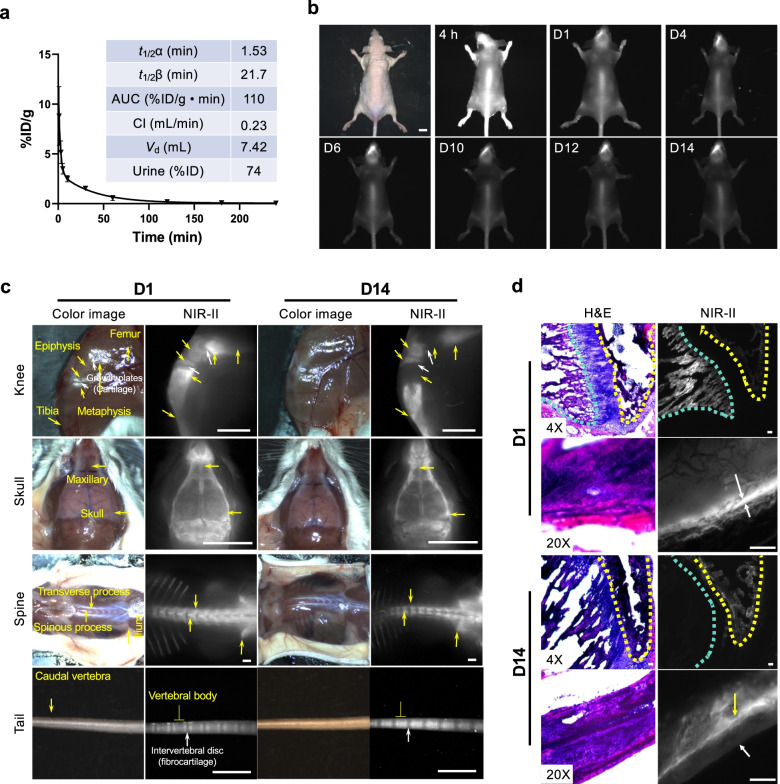
Stable incorporation of P800SO3-PEG into the bone matrix. **a** Blood curve and pharmacokinetic parameters obtained from CD-1 mice retro-orbitally injected with 25 nmol of P800SO3-PEG. **b** Longitudinal biodistribution of P800SO3-PEG fluorophores in nude mice. 50 nmol (2.5 mg kg^−1^) of P800SO3-PEG was injected intravenously to 20 g athymic NCr nu/nu mice and imaged for 14 d post-injection. Scale bars = 1 cm. SBR was calculated as the fluorescence intensity of each spine divided by the signal intensity of the neighboring skin as a background (*n* = 3, mean ± S.D). **c** Long-term signal integrity of P800SO3-PEG. 50 nmol of P800SO3-PEG was injected intravenously to 20 g CD-1 mice 1 d or 14 d prior to imaging (*n* = 3, mean ± S.D). Scale bars = 4 mm. **d** H&E and NIR imaging of resected bone tissues taken from mice 1 d and 14 d post-injection (**c**). Scale bars = 50 µm

Next, we evaluated the pharmacokinetics and excretion route of P800SO3-PEG (Fig. 4a). P800SO3-PEG quickly distributed into the whole body and cleared rapidly from the bloodstream (*t*_1/2_α = 1.53 min; *t*_1/2_β = 21.7 min), unlike P800SO3, which showed slower clearance (*t*_1/2_α = 7.4 and *t*_1/2_β = 51.6 min) [29] likely due to the lower Log*D* (-8.65) and flexible ethylene glycol units. Consequently, P800SO3-PEG showed a small area under the curve (AUC) and a reasonable clearance rate (0.23 mL/min), resulting in fast urinary excretion within 4 h (74%ID). To determine the dose-dependent bone targeting and biodistribution, 10–100 nmol (0.5–5 mg kg^−1^) of P800SO3-PEG were injected into CD-1 mice 4 h prior to imaging (Fig. S6).

### Noninvasive NIR-II imaging

In the NIR-II window, the skull, scapula, spine, ilium, and part of the femur bones were clearly detected in the tested dose ranges. The maximum average SBR value of 22.0 ± 1.3 was obtained at a dose of 50 nmol (***p < 0.001). Although 100 nmol of P800SO3-PEG amplified the bone signal, the SBR decreased due to the increased background signal (SBR ≈ 15). P800SO3-PEG was eliminated from the body by renal clearance into the urinary bladder without nonspecific uptake by non-bone tissues even at the highest dose of 100 nmol. This could be explained by the low LogD value (-8.65 at pH 7.4) and high polarity (TPSA = 294.30 Å^2^) of P800SO3-PEG.

To reduce the influence of light scattering, 50 nmol (2.5 mg kg^−1^) of P800SO3-PEG was injected intravenously into athymic NCr nu/nu mice, and their bone signals were imaged over the course of two weeks (Fig. 4b). The SBR of bones gradually increased and reached a peak of 4.80 at 4 d post-injection and maintained a signal of 3.5 for two weeks (Fig. S5f). Figure 4c compares the bone imaging of the knee, skull, spine, and tail in mice 1 d and 14 d post-intravenous injection of P800SO3-PEG. The magnified bone images show a shift of bone signal distributions in the tissue. In the knee joint, the epiphysis and metaphysis are metabolically active areas from which signals diminished or disappeared over time and redistributed into the tibia and femur bones. On the other hand, there was no signal in the growth plate since it is a cartilage tissue. As for the skull and maxillary, the edge profile signals became more apparent over time. The planform of the back trunk shows the spine (including thoracic, lumbar, and sacral vertebrae), ilium, and part of the dorsal ribs. Under the NIR-II window, the signals in the spinous processes gradually decreased while the signals in the transverse processes were retained. Figure 4c also shows precise tail vertebra imaging of an unskinned mouse. After two weeks of deposition, each caudal cone gave off a high signal intensity and could be visually separated. There is no fluorescent signal in the intervertebral disc since it is a fibrocartilage tissue. H&E and microscopic analysis of longitudinal bones on day 1 showed fluorescence signals on the bone surface (Fig. 4d) indicating cartilage (white arrows), and imaging on day 14 post-injection revealed that P800SO3-PEG was stably incorporated into the bone matrix (yellow arrows) with additional normal bone matrix deposited on top of the NIR fluorophores over time. Microscopic analysis of cancellous bone indicated that this region experienced high rates of turnover (i.e., the removal of P800SO3-PEG-labeled tissue (blue dashed line) and/or delayed new bone deposition (no fluorescence) [42].

### 3D fluorescence tomography imaging

**Fig. 5:**
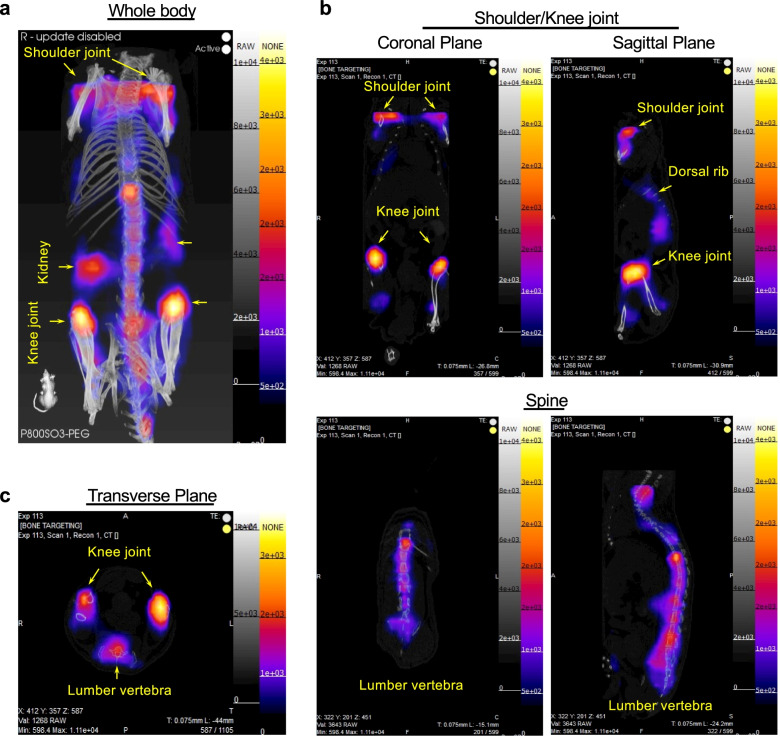
3D fluorescence tomography imaging of P800SO3-PEG in nude mice under the InSyTe FLECT/CT imaging system. 100 nmol (2.5 mg kg^−1^) of P800SO3-PEG was injected into athymic NCr *nu/nu* mice 4 d prior to imaging. **a** Whole-body, **b** coronal and sagittal, and **c** transverse sections of fluorescence and CT images. FLECT/CT images were taken with a 780 nm laser excitation and an 853 nm NP filter

Computed tomography (CT) is the most common imaging method widely used to provide bone images at the macro and micro levels. Additionally, 3D fluorescence tomography provides the chance to see deep tissue NIR fluorescence imaging, due to the wide-angle (360°) acquisition of the fluorescence data [43]. Together, the fluorescence emission computed tomography (FLECT)/CT system allows the capture of authentic 3D tomographic images by acquiring projection images 360° around the animal subject and subsequent accurate image reconstruction [28]. Figure 5 and Video S1 show the in vivo distribution of P800SO3-PEG in various bone tissues by 3D fluorescence tomography under the InSyTe FLECT/CT imaging system. Compared with other tissues, P800SO3-PEG demonstrated an evident bone uptake at 4 d post-injection. Figure 5a shows the accumulation of P800SO3-PEG in the spine and bone joints. This data also indicates that P800SO3-PEG preferentially accumulated in the metabolically active bone regions. The residual weak kidney signal indicates the steady renal clearance of P800SO3-PEG. The coronal and sagittal images also show a substantial accumulation of P800SO3-PEG in the knee, shoulder, and spine (Fig. 5b,c).

## Discussion

Bisphosphonates display a high affinity for calcium on the surface of HA and CP in the bone [44, 45]. Armed with the SIT design strategy, we incorporated the targeting moiety of phosphonates into the non-delocalized structure of heptamethine indocyanines to create a series of bone-targeted NIR fluorophores. Particularly, P800SO3-PEG shows fast tissue distribution (*t*_1/2_α = 1.53 min), minimum nonspecific uptake (AUC = 110%ID/g ⋅ min; Cl = 0.23 mL/min), and rapid renal clearance (74%ID, 4 h) after intravenous injection. The PEG linker on the mesocarbon and the sulfonates on the indole rings improve the solubility in aqueous media (> 100 mg/mL). Together, this bifunctional bone-targeted agent created by the SIT design strategy reduces plasma protein binding and off-target distribution during systemic circulation, thus increasing targetability to the bone tissue [22, 29, 45].

This SIT design strategy also affects the optical properties of the final fluorophores. Compared to P800SO3, the other three fluorophores showed decent NIR-II signals in 10% FBS when imaged under the 1,070 nm LP filter (Fig. 2a). This proves that the thiol-PEG substitution on the meso-carbon of the heptamethine core has proven effective in increasing the maximum absorbance wavelength and the bathochromic fluorescence emission, enhancing the fluorescence emission intensity in the NIR-II window. Compared to NIR-I imaging, NIR-II imaging using P800SO3-PEG can provide more precise information with higher resolution, particularly in deep bone tissues. The results of 3D fluorescence tomography imaging indicate that even the current maximum fluorescence wavelength detected by the FLECT/CT system is 900 nm, and the fluorescence emission of P800SO3-PEG is still mainly in the NIR-I region (Fig. 5 and Video S1). However, P800SO3-PEG shows an increased λmax as the energy decreases, enabling the extension of the fluorescence tail into the NIR-II window, representing its potential in non-invasive and non-radiative real-time bone imaging.

Interestingly, the introduction of thiol on the mesocarbon contributed to the photothermal effect of the NIR fluorophores, where P800SO3-SH and P800SO3-PEG exhibited rapid photodegradation within 1 h of laser exposure (Fig. S3a). This result is consistent with the known photothermal effect of ZW800-PEG, where the incident photons are absorbed and converted into heat energy [33–35]. The thiol-PEG linker is physiochemically stable yet photosensitive to NIR light, generating photothermal effects by incorporating excited state singlet oxygen into the polymethine backbone leading to decomposition and the production of carbonyl-containing fragments (Fig. 1h). The reduced QY of P800SO3-PEG (7.34%) compared to P800SO3 (15.1%) is mainly due to the energy loss from the photothermal effect, making P800SO3-PEG a promising photothermal theranostic agent.

Overall, P800SO3-PEG has a highly efficient and quick binding to the bone matrix and relatively fast renal clearance due to the balanced skeleton composed of bisphosphonates and sulfonates along with the flexible thiol PEG linker. P800SO3-PEG has several advantages compared to our previously reported bone-targeted agent P800SO3 and other bisphosphonate-based NIR fluorescent contrast agents: 1) improved bone-specific targeting with higher SBR, 2) maximum absorption and emission spectra around 800 nm, compatible with conventional NIR imaging systems, 3) improved noninvasive imaging under the NIR-II window, and 4) thiol-induced photothermal therapy for bone tumors, metastases, and infectious diseases [46, 47]. Additionally, unlike typical NIR-II fluorescent nanoparticles [48, 49], which are primarily retained in the body or show slow hepatobiliary excretion, renal clearable P800SO3-PEG shows rapid excretion and good biocompatibility with no interference in the liver, spleen, and other organs.

## Conclusions

Bone-targeted NIR fluorophores have significant clinical implications for image-guided cancer surgery and the treatment of bone diseases. First, intraoperative NIR fluorescence imaging can differentiate the tumors from nearby normal tissues by visualizing them separately with two different colors. Since the bone-targeted NIR fluorophores are mostly 800 nm emitting, the combination of 700 nm or over 1,000 nm emitting fluorophores targeting other tissues, including cartilage [50], tendon [27], nerve [51], vasculature [25], and inflammation [28], enables real-time multispectral imaging and/or the prognostic evaluation of bone metastasis. Second, the photothermal effect of P800SO3-PEG with the features of noninvasiveness and high selectivity makes it useful as a promising adjunct and/or alternative technique to traditional cancer treatments [52, 53]. Its subsequent applications may include but are not limited to the treatment of osteolytic lesions that occur in all bone cancer patients. Lastly, bisphosphonates are widely used to prevent bone loss and hypercalcemia by inhibiting calcification and suppressing bone resorption, thereby increasing bone strength and treating osteoporosis [44, 45]. These clinical applications of bone-targeted contrast agents can be elucidated by multimodal imaging of 2D/3D NIR fluorescence and CT.

In summary, we report that P800SO3-PEG is a bone-targeted heptamethine cyanine fluorophore for theranostic imaging. Due to the balanced skeleton composed of bisphosphonates and sulfonates along with the flexible thiol PEG linker, P800SO3-PEG shows a high affinity for bone tissues, deeper tissue imaging capabilities, minimum nonspecific uptake in the major organs, and photothermal effect upon laser irradiation. P800SO3-PEG is an optimal bone-targeted theranostic agent for both NIR-I and NIR-II imaging with solid promise for clinical translation.

## Supplementary Information

**Additional file 1: Video S1.** Fluorescence tomography imaging of P800SO3-PEG in a nude mouse.

**Additional file 2: **Of synthetic methods, LC-MS and NMR-spectra, photostability, photothermal effect, electron density calculations, plasma protein binding assays, microscopic image, biodistribution, dose-dependent bone-specific targeting, and fluorescence tomography imaging. **Fig. S1**. LC-MS analyses of P800SO3-Cl, P800SO3-SH, and P800SO3-PEG. **Fig. S2.** ^1^H and ^13^C NMR spectroscopy of P800SO3-Cl, P800SO3-SH, and P800SO3-PEG. **Fig. S3.** Photostability and physicochemical properties of bone-targeted NIR fluorophores. **Fig. S4.** Microscopic images of P800SO3-PEG binding to various calcium salts. **Fig. S5.** Biodistribution and bone-specific targeting of NIR fluorophores in mice. **Fig. S6.** Dose-dependent bone-specific targeting of P800SO3-PEG in mice.

## Data Availability

The datasets and materials used and/or analyzed during the current study are available from the corresponding author on reasonable request.
