# Supplementary figures and images for: P800SO3-PEG: a renal clearable bone-targeted fluorophore for theranostic imaging
Source: Biomater Res. 2022 Oct 1;26:51. doi: 10.1186/s40824-022-00294-2 (PMC9526902; doi:10.1186/s40824-022-00294-2)

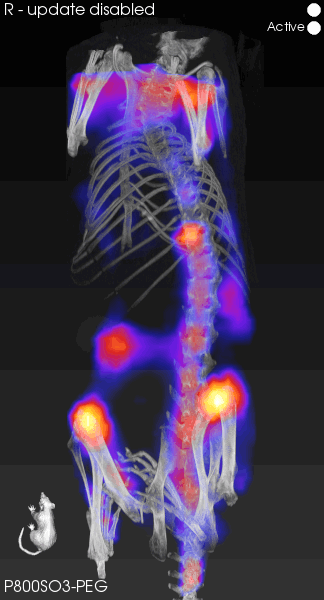

Supplement: Supplementary file 1 — Additional file 1: Video S1. Fluorescence tomography imaging of P800SO3-PEG in a nude mouse. [file 40824_2022_294_MOESM1_ESM.gif]
