# Supplementary material for: P800SO3-PEG: a renal clearable bone-targeted fluorophore for theranostic imaging
Source: Biomater Res. 2022 Oct 1;26:51. doi: 10.1186/s40824-022-00294-2 (PMC9526902; doi:10.1186/s40824-022-00294-2)
Supplement: Supplementary file 2 — Additional file 2: Of synthetic methods, LC-MS and NMR-spectra, photostability, photothermal effect, electron density calculations, plasma protein binding assays, microscopic image, biodistribution, dose-dependent bone-specific targeting, and fluorescence tomography imaging. Fig. S1. LC-MS analyses of P800SO3-Cl, P800SO3-SH, and P800SO3-PEG. Fig. S2. 1H and 13C NMR spectroscopy of P800SO3-Cl, P800SO3-SH, and P800SO3-PEG. Fig. S3. Photostability and physicochemical properties of bone-targeted NIR fluorophores. Fig. S4. Microscopic images of P800SO3-PEG binding to various calcium salts. Fig. S5. Biodistribution and bone-specific targeting of NIR fluorophores in mice. Fig. S6. Dose-dependent bone-specific targeting of P800SO3-PEG in mice. [file 40824_2022_294_MOESM2_ESM.pdf]

<u>**Supplementary Information**</u>

P800SO3-PEG: a renal clearable bone-targeted fluorophore for theranostic imaging

Haoran Wang[1,2], Homan Kang[2], Jason Dinh[2], Shinya Yokomizo[2], Wesley R. Stiles[2], Molly Tully[2], Kevin Cardenas[2], Surbhi Srinivas[2], Jason Ingerick[2], Sung Ahn[2], Kai Bao[2*], and Hak Soo Choi[2*]

[1]Wuya College of Innovation, Shenyang Pharmaceutical University, Shenyang 110016, China

[2]Gordon Center for Medical Imaging, Department of Radiology, Massachusetts General Hospital and Harvard Medical School, Boston, MA 02129, USA

[*]Correspondence to K.B. kbao@mgh.harvard.edu; H.S.C. hchoi12@mgh.harvard.edu

The PDF file includes:

Figure S1. LC-MS analyses of P800SO3-Cl, P800SO3-SH, and P800SO3-PEG.

Figure S2. ^{1}H and ^{13}C NMR spectroscopy of P800SO3-Cl, P800SO3-SH, and P800SO3-PEG.

Figure S3. Photostability and physicochemical properties of bone-targeted NIR fluorophores.

Figure S4. Microscopic images of P800SO3-PEG binding to various calcium salts.

Figure S5. Biodistribution and bone-specific targeting of NIR fluorophores in mice.

Figure S6. Dose-dependent bone-specific targeting of P800SO3-PEG in mice.

Video S1. Fluorescence tomography imaging of P800SO3-PEG in a nude mouse.

Synthesis of bone-targeted phosphonates.

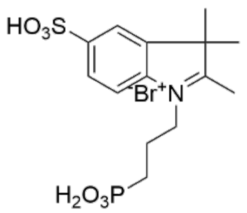

1

2,3,3-trimethyl-1-(3-phosphonopropyl)-5-sulfo-3H-indol-1-ium bromide **(1)**. A mixture of potassium 2,3,3-trimethylindolene-5-sulfonic acid (3 g, 4.2 mmol) and (3-bromopropyl) phosphonic acid (3 g, 5 mmol) in toluene (100 mL) was heated at 100°C for 72 h under a nitrogen atmosphere. The mixture was cooled to room temperature and the solvent was decanted. The crude mixture was filtered, collected, re-dissolved in a 1:1 (v/v) mixture of DW (4.2 mL) and MeOH (7.8 mL). The undissolved solid was removed by filtration. The mixture solution was then slowly added into ACN (210 mL) using a dropping funnel. The precipitate was filtered, dried, and checked by LC-MS. This product was collected as a red solid and used in the next step without further purification. (1.07 g, 22.7 %).[1]

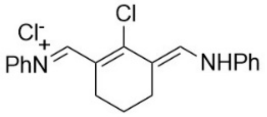

2

Compounds **2** was synthesized as previously reported by our group [2].

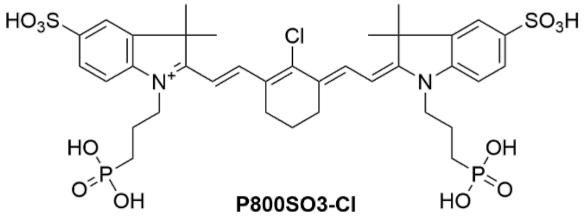

P800SO3-Cl

2-((E)-2-((E)-2-chloro-3-(2-((E)-3,3-dimethyl-1-(3-phosphonopropyl)-5-sulfoindolin-2-ylidene)ethylidene)cyclohex-1-en-1-yl)vinyl)-3,3-dimethyl-1-(3-phosphonopropyl)-5-sulfo-3H-indol-1-ium (**P800SO3-Cl**). A mixture of compound **1** (1 g, 2.7 mmol), Vilsmeier-Haack reagent

2 (0.45 g, 1.2 mmol), and anhydrous sodium acetate (0.3 g, 3.6 mmol) in absolute ethanol (20 mL) was heated under reflux for 6 h under a nitrogen atmosphere. The reaction mixture was cooled to room temperature, then dried by a rotovap to remove the reaction solvent, and finally washed with ethyl acetate and ethanol. **P800SO3-Cl** was further purified using Preparative HPLC by dissolving 100 mg of **P800SO3-Cl** in 2 mL of DW and sonication, followed by loading the solution into a XBridge Prep C18 OBD column. The eluent around 9 min was collected, concentrated by rotary evaporation, and dried under vacuum overnight. The final product was re-dissolved in 10 mL of DW, freeze-dried, protected from light, and stored in refrigeration until further use (**P800SO3-Cl**; 0.93 g, Yield: 91.0 %). Accurate mass TOF MS m/z $[M]^+$ calculated for $[C_{36}H_{44}ClN_2O_{12}P_2S_2]^+$ 857.15, found $[M+H]^+$ 858.76, respectively. ^1H NMR (500 MHz, DMSO-d_6) δ = 8.27 (d, *J*=13.8, 2H), 7.81 (s, 2H), 7.66 (d, *J*=8.2, 2H), 7.45 (d, *J*=8.4, 2H), 6.43 (d, *J*=14.2, 2H), 4.29 (d, *J*=8.5, 4H), 3.92 (s, 2H), 2.73 (d, *J*=6.5, 4H), 1.83 (dd, *J*=58.0, 23.2, 10H), 1.69 (s, 12H), 1.55 (s, 2H).

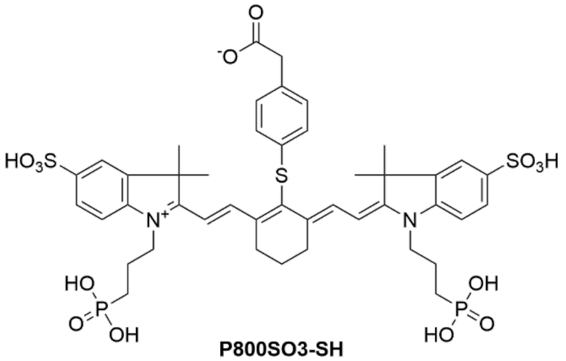

P800SO3-SH

2-(4-(((E)-2-((E)-2-(3,3-dimethyl-1-(3-phosphonopropyl)-5-sulfo-3H-indol-1-ium-2-yl)vinyl)-6-(2-((E)-3,3-dimethyl-1-(3-phosphonopropyl)-5-sulfoindolin-2-ylidene)ethylidene)cyclohex-1-en-1-yl)thio)phenyl)acetate (**P800SO3-SH**). **P800SO3-Cl** (100 mg, 0.16 mmol) was added into a 50-mL round bottom flask. Then, 5 mL of DMSO was added and the powder was dissolved totally by using sonication. DIEA (64 µL; 0.32 mmol; 2.0 molar equiv.) was pipetted into the flask and mixed well and 4-mercaptophenylacetic acid (30.3 mg; 0.18 mmol; 1.11 molar equiv.) was added into the reaction solution which was heated for 40 min at 60 °C in an oil bath. The reaction progress was checked using LC-MS system at the timepoint 40 min. After the reaction, the solution was

cooled down to room temperature then slowly poured into 50 mL of EA with 0.5 mL formic acid (0.1 v/v%) and stirred at room temperature for 30 min. The suspension was filtered to collect the precipitate, then it was washed by 10 mL EA twice. The precipitate was dried at ambient temperature overnight and collected as a dark green solid. **P800SO3-SH** was further purified using Preparative HPLC by dissolving 50 mg of **P800SO3-SH** in 2 mL of DW and sonication, followed by loading the solution into an XBridge Prep C18 OBD column. The eluent around 9.5 min was collected, concentrated by rotary evaporation, and dried under vacuum overnight. The final product was re-dissolve in 10 mL of DW, freeze-dried, protected from light, and stored in refrigeration until further use. (**P800SO3-SH**; 93.5 mg, Yield: 81.1%); Accurate mass TOF MS m/z $[M]^+$ calculated for $[C_{44}H_{52}N_2O_{14}P_2S_3]^+$ 990.21, found $[M+H]^+$ 991.86, respectively. ^{1}H NMR (500 MHz, DMSO-d_6) δ = 8.59 (d, *J*=14.0, 2H), 7.68 (s, 2H), 7.62 (d, *J*=8.1, 2H), 7.40 (d, *J*=8.5, 2H), 7.23 (s, 4H), 6.42 (d, *J*=14.3, 2H), 4.25 (d, *J*=7.9, 4H), 3.46 (s, 2H), 2.77 (t, *J*=6.2, 4H), 1.90 (q, *J*=7.4, 6H), 1.82 – 1.51 (m, 6H), 1.41 (s, 12H). ^{13}C NMR (150 MHz, DMSO-d_6) δ = 172.48, 172.33, 145.25, 145.11, 142.18, 140.62, 133.85, 132.93, 130.87, 126.24, 126.07, 119.81, 110.58, 102.27, 48.85, 43.89, 27.32, 26.00, 24.85, 23.94, 21.17.

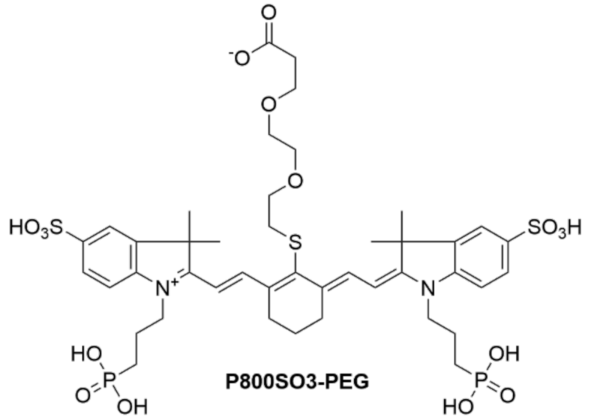

3-(2-(2-(((E)-2-((E)-2-(3,3-dimethyl-1-(3-phosphonopropyl)-5-sulfo-3H-indol-1-ium-2-yl)vinyl)-6-(2-((E)-3,3-dimethyl-1-(3-phosphonopropyl)-5-sulfoindolin-2-ylidene)ethylidene)cyclohex-1-en-1-yl)thio)ethoxy)ethoxy)propanoate (**P800SO3-PEG**). **P800SO3-Cl** (100 mg, 0.16 mmol) was added into a 50-mL round bottom flask . Then, 4 mL of DMSO was added and the powder was

dissolved totally by using sonication. DIEA (64 µL; 0.32 mmol; 2.0 molar equiv.) was pipetted into the flask and mixed well. Thiol-PEG (35.0 mg; 0.18 mmol; 1.11 molar equiv.) in 1 mL of DMSO was added into the reaction solution which was heated for 40 min at 60 °C in an oil bath.[2] The progress was checked using LC-MS system at the timepoint 40 min. After the reaction, the solution was cooled down to room temperature, then slowly poured into 50 mL of EA with 0.5 mL formic acid (0.1 v/v%), and stirred at room temperature for 30 min. The suspension was filtered to collect the precipitate, and lastly washed with 10 mL EA twice. The precipitate was dried at ambient temperature overnight and collected as a dark green solid. **P800SO3-PEG** was further purified using Preparative HPLC by dissolving 50 mg of **P800SO3-PEG** in 2 mL of DW and sonicating, followed by loading the solution into a XBridge Prep C18 OBD column. The eluent around 9.5 min was collected, concentrated by rotary evaporation, and dried under vacuum overnight. The final product was re-dissolve in 10 mL of DW, freeze-dried, protected from light, and stored in refrigeration until further use. (**P800SO3-PEG**; 98.4 mg, Yield: 83.1%); Accurate mass TOF MS m/z $[M]^+$ calculated for $[C_{43}H_{59}N_2O_{16}P_2S_3]^+$ 1016.24, found $[M+H]^+$ 1016.84, respectively. ^{1}H NMR (500 MHz, DMSO-d_6) δ = 8.75 (d, J=14.0, 2H), 7.79 (d, J=1.7, 2H), 7.65 (dd, J=8.1, 1.7, 2H), 7.41 (d, *J*=8.4, 2H), 6.41 (d, *J*=14.0, 2H), 4.27 (t, *J*=7.7, 4H), 3.64 – 3.47 (m, 8H), 3.46 (s, 4H), 2.98 (d, *J*=6.2, 2H), 2.66 (t, *J*=6.1, 4H), 2.46 – 2.38 (m, 4H), 1.92 (d, *J*=7.3, 4H), 1.81 (d, *J*=5.5, 2H), 1.71 (s, 12H). ^{13}C NMR (150 MHz, DMSO) δ = 172.58, 172.16, 145.23, 145.02, 142.26, 140.38, 133.48, 126.20, 119.91, 110.32, 101.75, 69.59, 69.42, 66.26, 48.86, 36.48, 34.75, 27.40, 25.77, 24.87, 23.96, 21.10, 20.62.

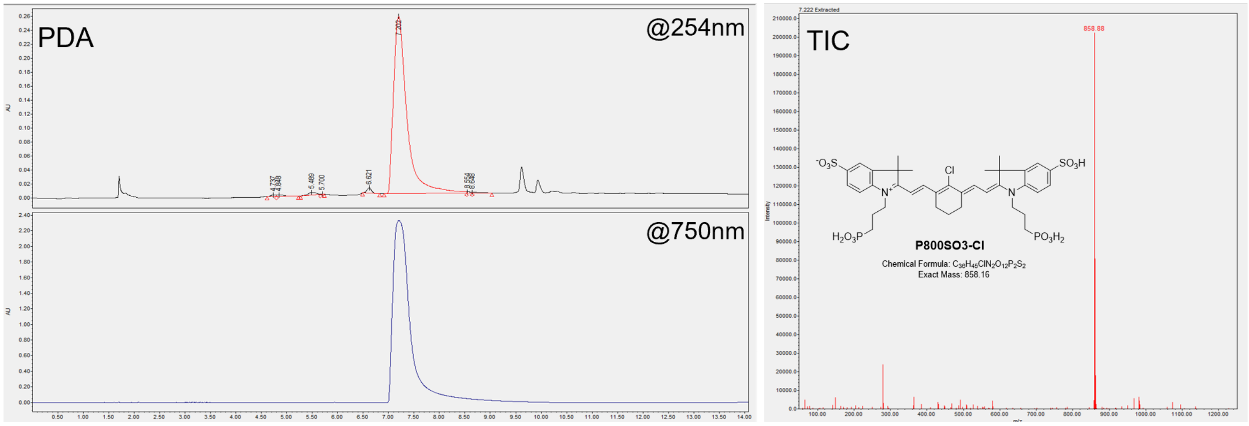

Figure S1a. LC-MS analysis of P800SO3-Cl. The mobile phase used was solvent A = 0.1% formic acid (FA) in water, solvent B = 0.1% FA in MeOH with a linear gradient from 5% to 95% (from A to B for 1 to 8 min), the flow rate was 1 mL/min, and the sample injection concentration was 1 mg/mL.

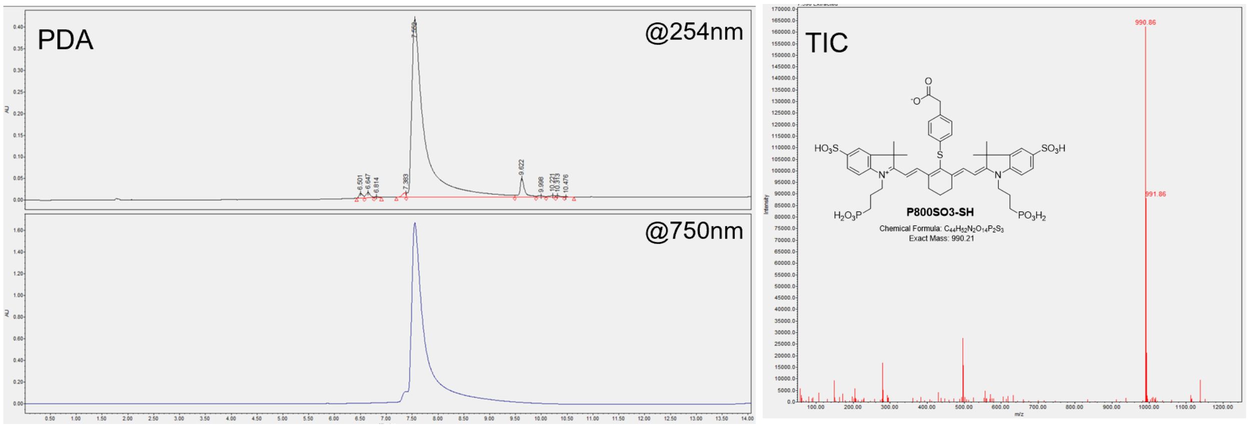

Figure S1b. LC-MS analysis of P800SO3-SH. The mobile phase used was solvent A = 0.1% formic acid (FA) in water, solvent B = 0.1% FA in MeOH with a linear gradient from 5% to 95% (from A to B for 1 to 8 min), the flow rate was 1 mL/min, and the sample injection concentration was 1 mg/mL.

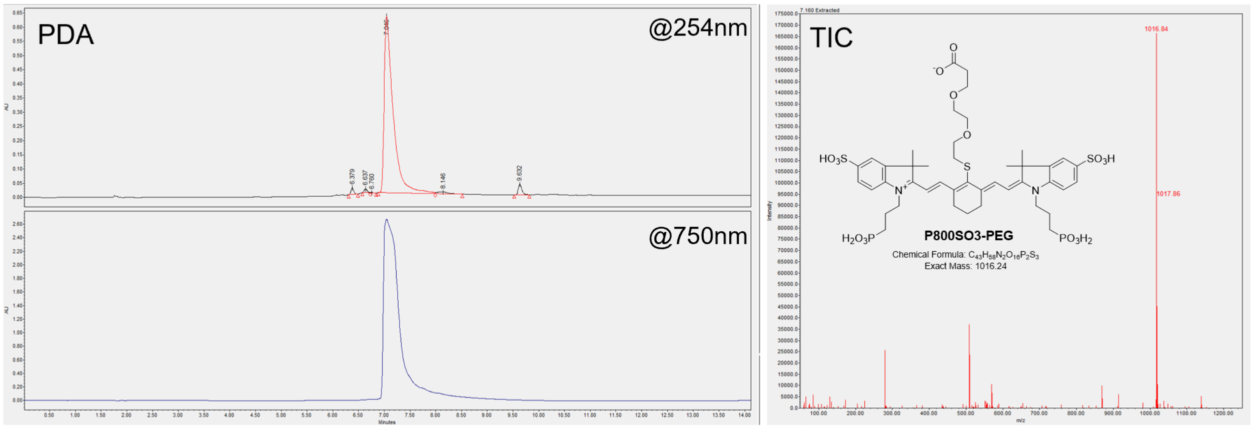

Figure S1c. LC-MS analysis of P800SO3-PEG. The mobile phase used was solvent A = 0.1% formic acid (FA) in water, solvent B = 0.1% FA in MeOH with a linear gradient from 5% to 95% (from A to B for 1 to 8 min), the flow rate was 1 mL/min, and the sample injection concentration was 1 mg/mL.

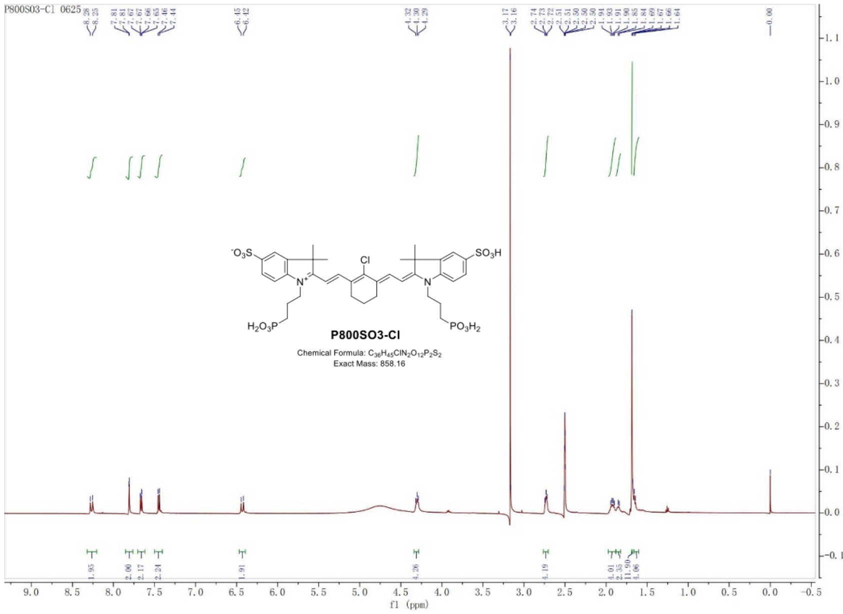

Figure S2a. ^{1}H-NMR spectroscopy of **P800SO3-Cl** in DMSO-d_6.

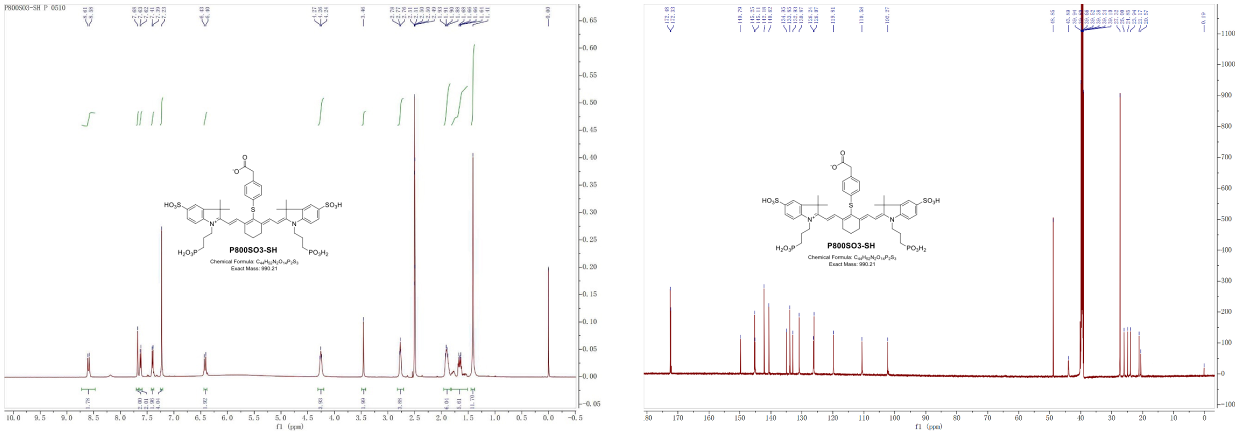

Figure S2b. ^{1}H and ^{13}C NMR spectroscopy of **P800SO3-SH** in DMSO-d_6.

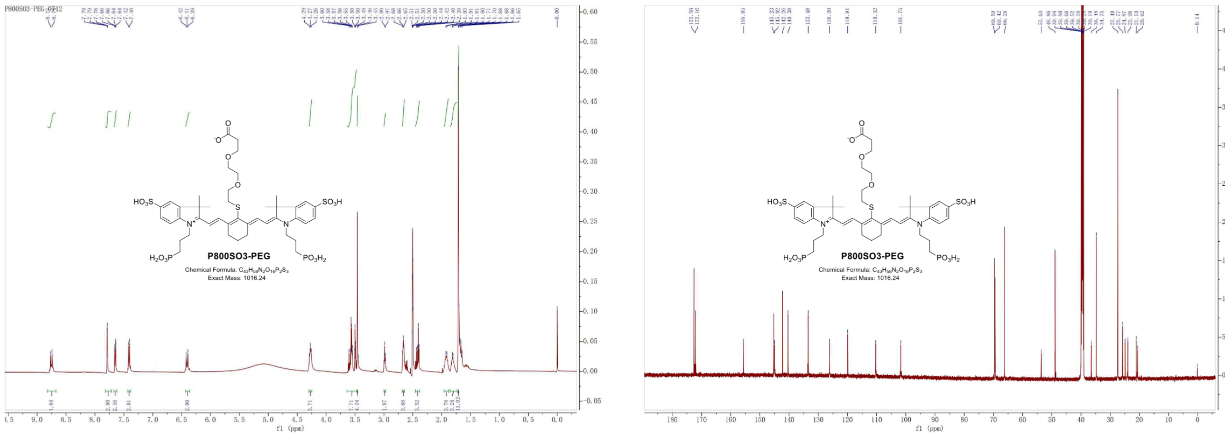

Figure S2c. ^{1}H and ^{13}C NMR characterization of **P800SO3-PEG** in DMSO-d_6.

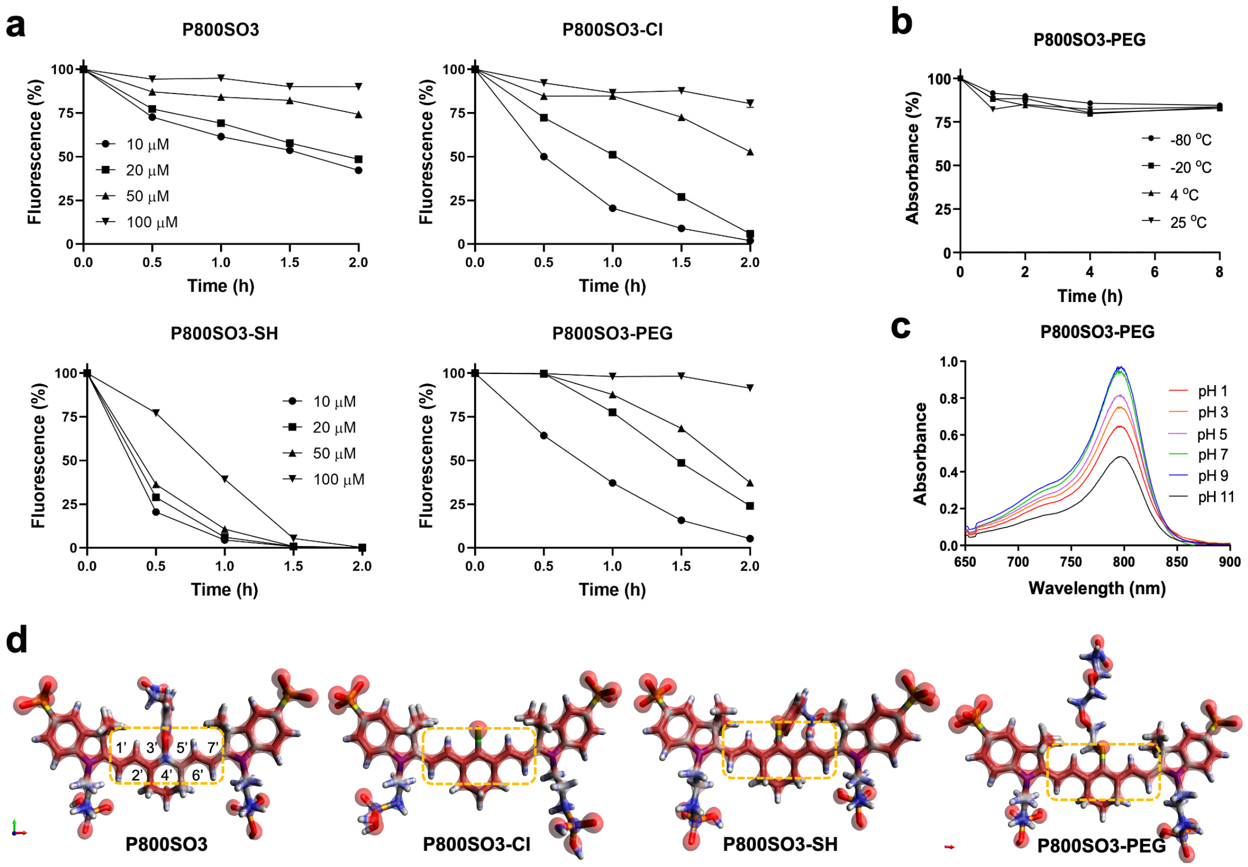

	1'	2'	3'	4'	5'	6'	7'	Sum
P800SO3	-0.251	-0.153	0.096	0.248	0.100	-0.154	-0.252	**-0.366**
P800SO3-Cl	-0.247	-0.166	0.142	-0.180	0.143	-0.165	-0.247	**-0.720**
P800SO3-SH	-0.246	-0.169	0.132	-0.215	0.129	-0.160	-0.252	**-0.781**
P800SO3-PEG	-0.253	-0.172	0.124	-0.203	0.131	-0.169	-0.248	**-0.790**

Figure S3. Photostability and physicochemical properties of bone-targeted NIR fluorophores. **a**) Photostability of bone-targeted NIR fluorophores under continuous 808 nm exposure for 2 h at a power density of 35 mW/cm^2. **b**) Thermal and **c**) pH stability of P800SO3-PEG. 1 mM and 5 μM working solutions were prepared in DI water for thermal and pH stability of P800SO3-PEG, respectively. **d**) Visualization of the electron density and summary of the Mulliken charge at each carbon in the heptamethine chain. Geometry optimization was performed on the Gaussian 16 software. Electron density information was used to calculate the surfaces (isovalue = 0.2) on Avogadro 1.2.0. The yellow rectangles outline the heptamethine chains of the bone-targeted agents.

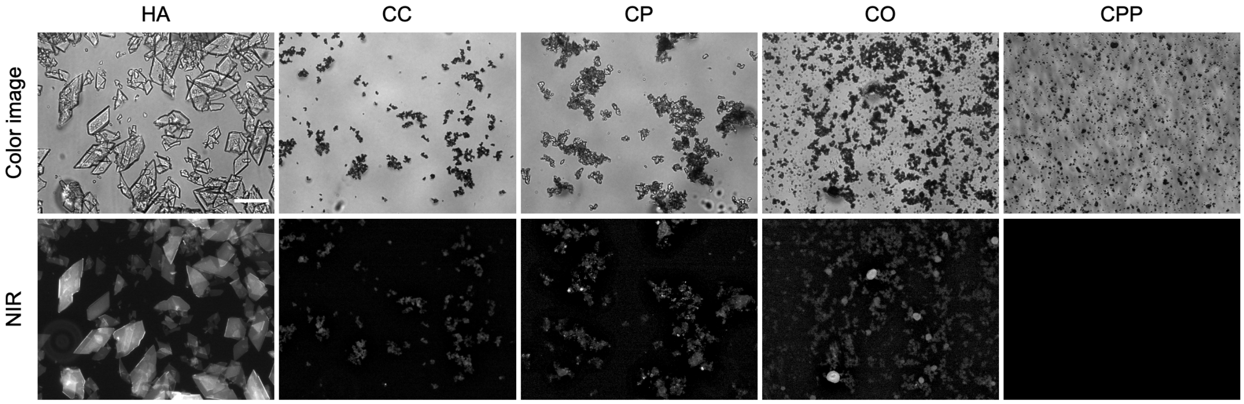

Figure S4. Microscopic images of P800SO3-PEG binding to various calcium salts. NIR fluorescence images for each condition have identical exposure times and normalization. Scale bar = 100 μm.

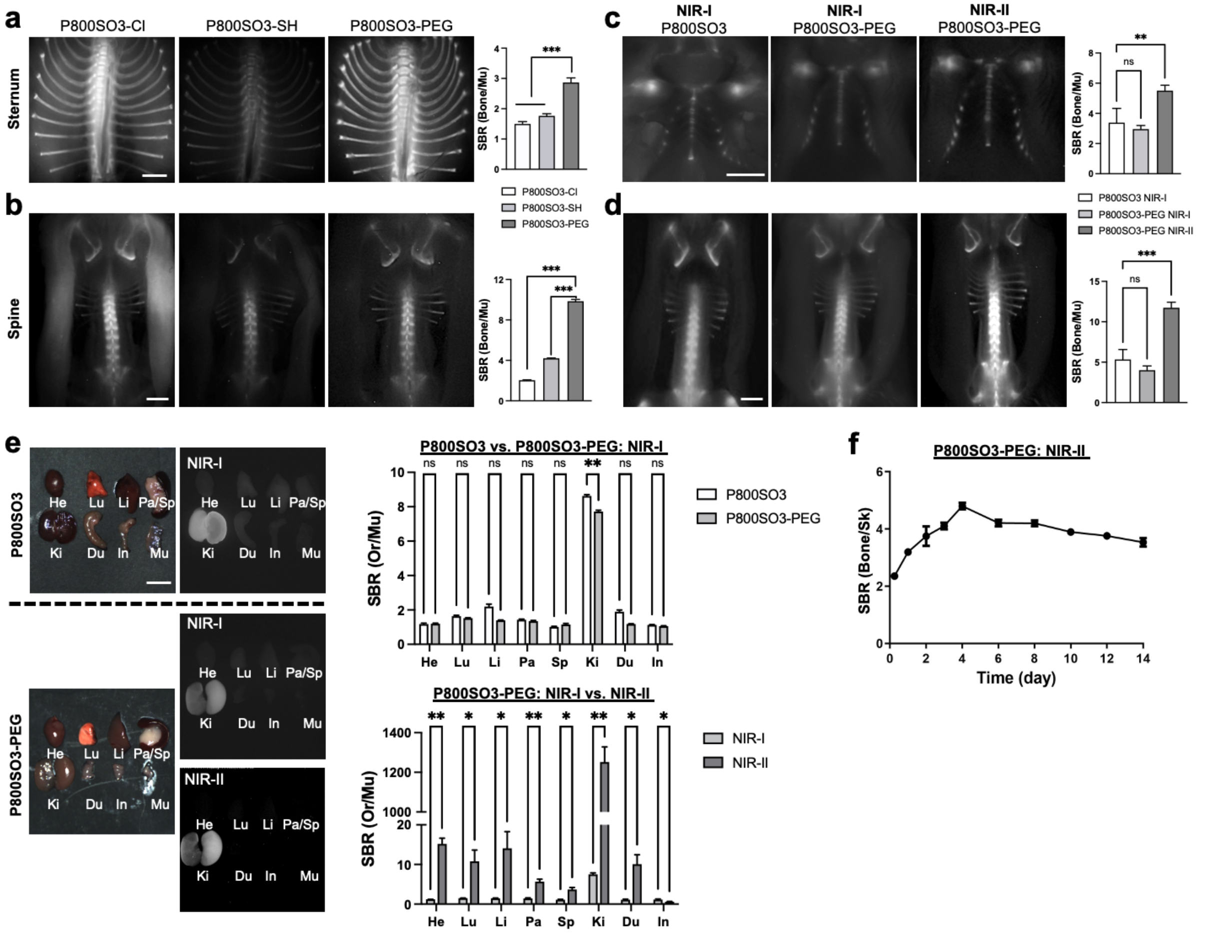

Figure S5. Biodistribution and bone-specific targeting of NIR fluorophores in mice. 20-50 nmol of each NIR fluorophore was injected intravenously to 20 g CD-1 mice 4 h prior to imaging. NIR fluorescence images for each condition have identical exposure times and normalization. **a,b**) Shown are NIR-II imaging of thoracic vertebrae (**a**) and spine (**b**) with P800SO3-Cl, P800SO3-SH, and P800SO3-PEG. Scale bars = 0.2 cm. Signal-to-background ratio (SBR) was calculated by the fluorescence intensity of each bone divided by the signal intensity of neighboring muscle (Mu) as background (n = 3, mean ± S.D.): ***p <0.001. **c-f)** Comparison of P800SO3 and P800SO3-PEG in the sternum and spine region under the NIR-I and NIR-II window. Shown are representative images of thoracic vertebrae (**c**), spine (**d**), resected organs (**e**), and their SBR compared to Mu (n = 3, mean ± S.D.): *ns*, not significant; *p <0.05; **p <0.01; ***p <0.001. Abbreviations used are: He, heart; Lu, lungs; Li, liver; Pa, pancreas; Sp, spleen; Ki, kidneys; Du, duodenum; In, intestine; Mu, muscle. Scale bars = 1 cm. **f)** Kinetics of P800SO3-PEG. 50 nmol (2.5 mg/kg) of P800SO3-PEG was injected intravenously to 20 g athymic NCr nu/nu mice and imaged for 14 d post-injection. SBR was calculated as the fluorescence intensity of each spine divided by the signal intensity of the neighboring skin as displayed in **Fig. 4b** (n = 3, mean ± S.D).

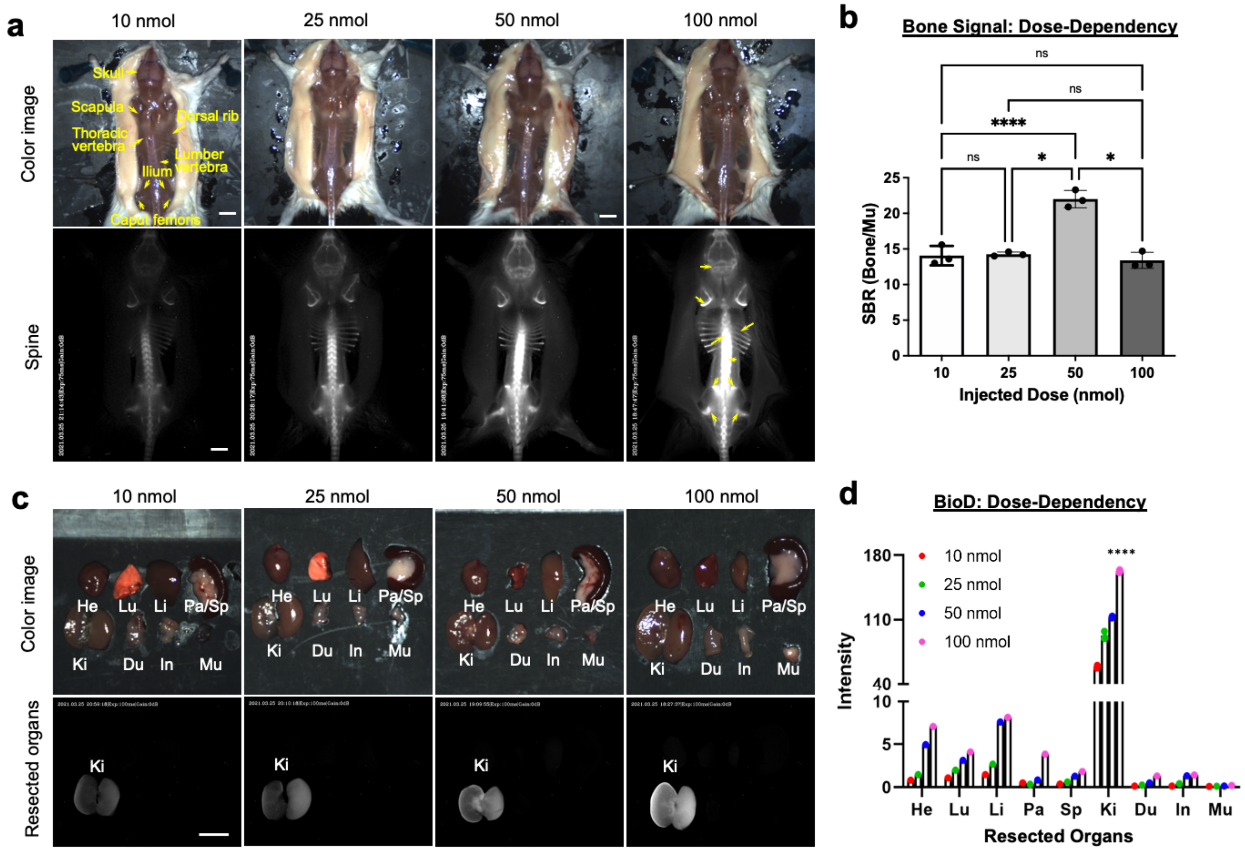

Figure S6. Dose-dependent bone-specific targeting of P800SO3-PEG in mice. 10-100 nmol (0.5-5 mg kg^{-1}) of P800SO3-PEG was injected intravenously into 20 g CD-1 mice 4 h prior to imaging. Real-time NIR-II imaging of spinal vertebrae (**a,b**) and resected organs (**c,d**) with P800SO3-Cl, P800SO3-SH, and P800SO3-PEG. SBR was calculated by the fluorescence intensity of the spine divided by the signal intensity of neighboring skin obtained over the imaging period. Abbreviations used are: Du, duodenum; He, Heart; In, intestine; Ki, kidneys; Li, liver; Lu, lungs; Mu, muscle; Pa, pancreas; Sp, spleen. NIR fluorescence images for each condition have identical exposure times and normalization (n = 3, mean ± S.D.): *ns*, not significant (p ≥0.05); *p <0.05; ****p <0.0001. Scale bars = 1 cm.

P800SO3-PEG
3D, D4

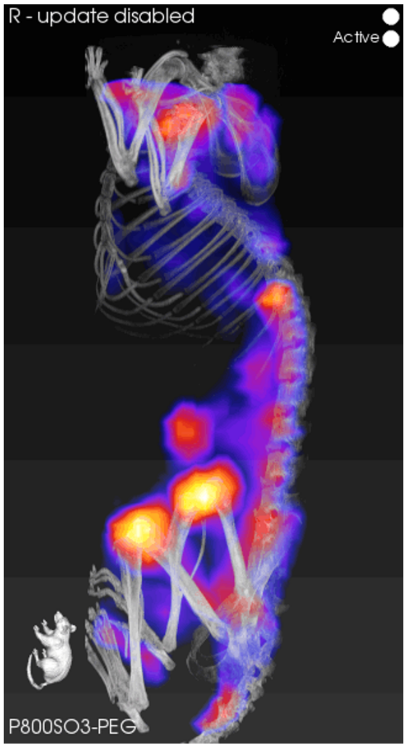

Video S1. Fluorescence tomography imaging of P800SO3-PEG in a nude mouse.

References

[1] Hyun H, Wada H, Bao K, Gravier J, Yadav Y, Laramie M, Henary M, Frangioni JV, Choi HS: **Phosphonated near-infrared fluorophores for biomedical imaging of bone**. *Angew Chem Int Ed Engl* 2014, **53**(40):10668-10672.

[2] Yang C, Wang H, Yokomizo S, Hickey M, Chang H, Kang H, Fukuda T, Song MY, Lee SY, Park JW *et al*: **ZW800-PEG: a renal clearable zwitterionic near-infrared fluorophore for potential clinical translation**. *Angew Chem Int Ed Engl* 2021, **60**(25):13847-13852.
